# Hospitalization causes and outcomes in HIV patients in the late antiretroviral era in Colombia

**DOI:** 10.1186/s12981-017-0186-3

**Published:** 2017-11-13

**Authors:** María Fernanda Álvarez Barreneche, Carlos Andrés Restrepo Castro, Alicia Hidrón Botero, Juan Pablo Villa Franco, Ivan Mauricio Trompa Romero, Laura Restrepo Carvajal, Alejandro Eusse García, Adriana Ocampo Mesa, Lina María Echeverri Toro, Glenys Patricia Porras Fernández de Castro, Jaime Mauricio Ramírez Rivera, Carlos Andrés Agudelo Restrepo

**Affiliations:** 1 School of Health Sciences, Universidad Pontificia Bolivariana, Carrera 44 # 18-56, Antioquia, Medellín, Colombia; 2 Division of Infectious Diseases, Department of Internal Medicine, IPS Universitaria Clínica León XIII, Antioquia, Medellín, Colombia; 3 Division of Infectious Diseases, Department of Internal Medicine, Hospital Pablo Tobón Uribe, Antioquia, Medellín, Colombia; 4 Division of Infectious Diseases, Department of Internal Medicine, Emory University School of Medicine, Atlanta, GA USA; 5 Division of Infectious Diseases, Department of Internal Medicine, Centros Especializados San Vicente Fundación, Antioquia, Rionegro, Colombia; 6 Division of Infectious Diseases, Department of Internal Medicine, Hospital Universitario, San Vicente Fundación, Antioquia, Medellín, Colombia; 7 Department of Internal Medicine, Hospital General de Medellín, Antioquia, Medellín, Colombia; 8 Division of Infectious Diseases, Department of Internal Medicine, Clínica Universitaria Bolivariana, Antioquia, Medellín, Colombia

**Keywords:** Acquired immune deficiency syndrome, Human immunodeficiency virus, Hospitalization, Opportunistic infections, Antiretroviral, Adherence

## Abstract

**Background:**

Antiretroviral therapy (ART) has modified the natural history of HIV-infection: the incidence of opportunistic infections (OIs) has decreased and mortality associated to HIV has improved dramatically. The reasons for hospitalization have changed; OIs are no longer the most common reason for admission. This study describes the patient population, admission diagnosis and hospital course of HIV patients in Colombia in the ART era.

**Methods:**

Patients admitted with HIV/AIDS at six hospitals in Medellin, Colombia between August 1, 2014 and July 31, 2015 were included. Demographic, laboratory, and clinical data were prospectively collected.

**Results:**

551 HIV-infected patients were admitted: 76.0% were male, the median age was 37 (30–49). A new diagnosis of HIV was made in 22.0% of patients during the index admission. 56.0% of patients of the entire cohort had been diagnosed with HIV for more than 1 year and 68.9% were diagnosed in an advanced stage of the disease. More than 50.0% of patients had CD4 counts less than 200 CD4 cells/μL and viral loads greater than 100,000 copies. The main reasons for hospital admissions were OIs, tuberculosis, esophageal candidiasis and Toxoplasma encephalitis. The median hospital stay was 14 days (IQR 8–23). Admission to the intensive care unit (ICU) was required in 10.3% of patients and 14.3% were readmitted to the hospital; mortality was 5.4%.

**Conclusions:**

Similar to other countries in the developing world, in Colombia, the leading cause of hospitalization among HIV-infected patients remain opportunistic infections. However, in-hospital mortality was low, similar to those described for high-income countries. Strategies to monitor and optimize the adherence and retention in HIV programs are fundamental to maximize the benefit of ART.

## Background

Life expectancy for patients infected with the human immunodeficiency virus (HIV) has improved significantly in the era of antiretroviral therapy (ART), largely due to the reduction in mortality attributable to diseases related to acquired immunodeficiency syndrome (AIDS) [1]. A recent systematic review and meta-analysis has summarized data on causes of hospital admission among children and adults living with HIV globally: AIDS-related illnesses (including tuberculosis [TB]) and bacterial infections were the second most common cause of adult HIV admissions in all geographical regions and the most common cause of hospital mortality [2]. Studies in high-income countries have shown changes in the main causes of hospitalization and death of HIV patients: opportunistic diseases have given way to chronic diseases and neoplasms not associated with AIDS [2–5]. On the other hand, in developing countries, high mortality rates persist despite availability of antiretrovirals; AIDS defining events continue to be reported as the main cause of hospitalization and death [6–10].

Although the change in the epidemiological profile of hospitalized patients with HIV has been widely documented in high-income and in developing countries, little is known about the reasons for hospital admission and hospital outcomes in the current era of the HIV epidemic in Colombia [11, 12]. This study describes the clinical characteristics, causes of hospital admission and mortality rates of patients with HIV infection from six high complexity care hospitals in Medellin, Colombia.

## Methods

### Study design and patient collection

Patients diagnosed with HIV, older than 18 years of age and hospitalized for more than 24 h in any of six reference hospitals in Medellin, Colombia, between August 1, 2014 and July 31, 2015, were included. Patients who were unconscious, pregnant women (who are usually treated in high risk obstetric units in Colombia, not in the infectious disease ward) and patients who did not agree to participate were excluded. The information was prospectively collected using Magpi^®^ software (formerly DataDyne, Washington, WA), and included socio-demographic variables, baseline characteristics at admission, reason for hospital admission and clinical outcomes. Patients that could not be interviewed were excluded from the analysis, but the reason for admission was recorded. The definitions of the variables were based on previous studies and those established by National Institutes of Health [13]. Adherence measurement was given by self-report of the patient: the patient taking more than 95% of total doses in the last month was defined as adherent according with Simoni et al. [14]. Hospital readmission was defined as a new (different) hospitalization for the same cause during the year of the study. Laboratory data and clinical outcomes were extracted from the patient´s medical chart.

### Ethics statement

The study was approved by the ethics committee of each of the participating hospitals. All study participants provided a written informed consent for collecting information; the patient´s identity was protected, individual records were coded and accessed only by research staff. None of the enrolled participants were below 18 years of age. A post hoc authorization was given to review the clinical records of all HIV patients hospitalized at participating hospitals during the study period in order to identify patients lost due to care in services other than infectious diseases.

### Statistical analysis

Data were exported to SPSS^®^ version 17 (IBM, Armonk, NY) to perform analyses using frequency distribution measures for qualitative variables and measures of central tendency, dispersion and position for quantitative variables. Manual review of medical records was performed to resolve discrepancies found in the database.

## Results

**Fig. 1:**
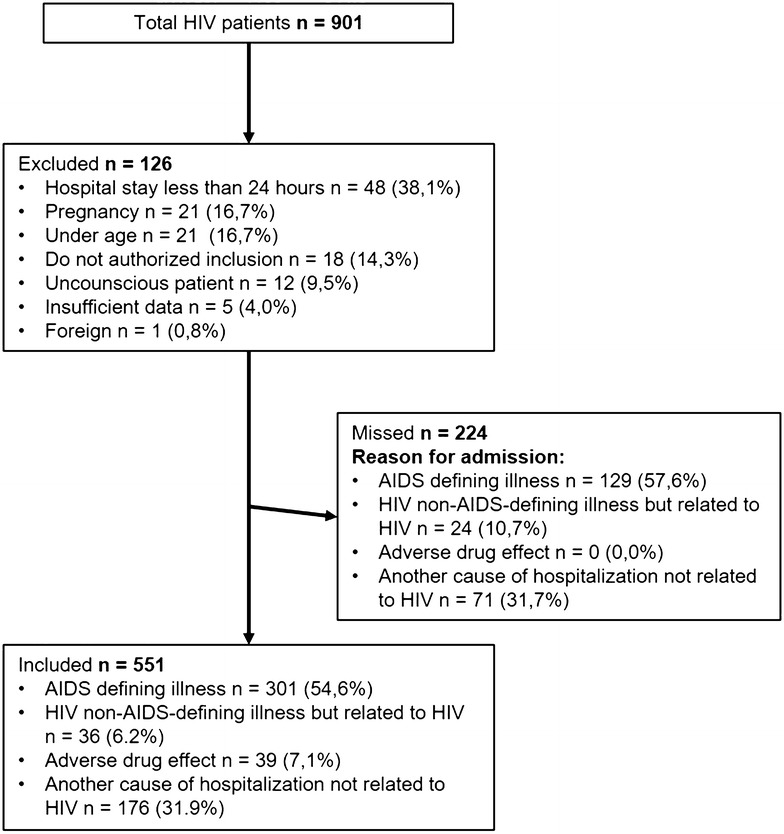
HIV patients hospitalized in Medellín, Colombia from August 2014 to July 2015

901 HIV-positive patients were hospitalized during the year of the study in participating institutions, representing 0.79% of 114.505 admissions. Of those, 551 patients were included. One hundred twenty-six (14%) patients were excluded and 224 (24.9%) patients were missed, mainly because they were cared for by services other than infectious diseases (Fig. 1).

The median age was 37 years (IQR 30–49). Patients mostly belonged to a low socioeconomic class (n = 372, 67.5%) with a basic education level (only elementary and/or high school) in 70.8% (390 patients). HIV diagnosis was made in advanced stage disease (< 200 CD4 cells/μL and/or AIDS defining entities) in 68.9% (380) of the patients. More than 50.0% of patients had CD4 counts less than 200 CD4 cells/μL and viral loads greater than 100,000 copies at admission. Diagnosis of HIV was made during the index admission in 22% (121) of patients and their median CD4 count was 59 cells/μL (interquartile range [IQR] 27–149.5). Of the 430 patients with a previous HIV diagnosis, 42% (181 patients) had already had an opportunistic infection, with tuberculosis being the most common one. In this group, ART had been prescribed to 85.1% (366/430), but only 63.4% (273/430) were receiving it on admission, and only 199 patients (54.1%) reported being adherent to ART. The main reasons for non-adherence were drug dependence, social or occupational instability, psychiatric disorders and lack of family support. Multiple antiretroviral regimens have been received by 48% (175/366) of patients; the average number of previous regimens was 1.5 (SD ± 0.82).

Baseline characteristics were similar for all patients compared to the subsets of patients with a previous HIV diagnosis and a prior AIDS-defining illness, except the median CD4 count was lower and the proportion of patients without antiretroviral therapy was greater in the AIDS-defining illness group (Table 1).

**Table 1:** Baseline characteristics for 551 patients with HIV infection admitted to medical ward

Patient characteristics	All patients, n = 551 (100%)	Previous HIV diagnosis, n = 430 (78%)	AIDS-defining illness, n = 301 (54.6%)
Age, median (IQR)	37 (30–49)	36 (30–48)	37 (30–50)
Male	418 (76)	321 (74.6)	231 (76.7)
Low income	372 (67.5)	289 (67.2)	225 (74.7)
Lower educational attainment	390 (70.8)	188 (43.7)	154 (51.1)
New HIV diagnosis	121 (22)	–	88 (29.2)
ART status			
Receiving ART prior to admission	273 (49.5)	273 (63.4)	131 (43.5)
Not on ART	278 (50.4)	64 (14.9)	111 (36.8)
Abandonment of ART	–	93 (21.6)	59 (19.6)
Adherence to ART^a^	–	199/366 (54.4)	83/190 (43.6)
Attending HIV program	–	258 (60.0)	86 (28.5)
Attending HIV program and receiving ART	–	226/258 (87.6)	72(23.9)
CD4 cell count, cells/μl Median (IQR)	98 (36.2–98)	117 (41–310)	59 (24–131)
HIV viral load, copies/mL Median (IQR)	100.278 (1.505–384.250)	36.381 (77–216.441)	179.000 (26.597–622.022)
Anemia^b^	104 (18.9)	90 (20.9)	76 (25.2)
Past history of TB	88 (20.4)	88 (20.4)	55 (18.2)

^a^Adherence to ART is calculated with patients who had abandoned ART or were receiving ART prior to admission

^b^Anemia was defined using WHO criteria (hemoglobin < 11.0 g/dL for both males and females)

The main reason for hospitalization was an AIDS-defining illness in 54.6% (301/551) of patients, and tuberculosis was the most frequent OI in this group (42.5%, 128/301). The second most frequent reason for admission were non HIV-related affections with bacterial infections predominating (11.0%, 62 of 551 hospitalizations) (Table 2).

**Table 2:** Causes of hospitalization in HIV patients in Medellín, Colombia from August 2014 to July 2015 (N = 551)

Variable	n (%)
AIDS defining illness^a^	301 (54.6)
Tuberculosis	128 (23.3)
Extrapulmonary tuberculosis	84 (15.2)
Pulmonary tuberculosis	44 (8.0)
Esophageal candidiasis	56 (10.1)
Toxoplasma encephalitis	43 (7.8)
Disseminated histoplasmosis	39 (7.1)
CMV infection	36 (6.5)
Extrapulmonary cryptococcosis	32 (5.8)
Pneumonia by *P. jiroveci*	32 (5.8)
Lymphoma^b^	27 (4.9)
Kaposi’s sarcoma	11 (2.0)
Herpes simplex > 1 month	10 (1.8)
Cryptosporidiosis > 1 month	10 (1.8)
HIV Encephalopathy	8 (1.4)
Wasting syndrome	7 (1.3)
MAC	4 (0.7)
Recurrent salmonellosis	2 (0.3)
Adverse drug effect	39 (7.1)
Non-AIDS-defining illness but related to HIV	35 (6.2)
Another cause of hospitalization not related to HIV	176 (31.9)
Bacterial infection	61 (11.0)
Others^c^	115 (20.9)

^a^The total percentage exceeds 100% because *some patients had* > *1 defining illness*

^b^Burkitt lymphoma, *primary central nervous system lymphoma, immunoblastic lymphoma*

^c^Other: gastrointestinal, neurological, hematological and cardiac alterations, viral infection, pneumopathy, reactive lymphadenopathy

Admission to the ICU was required in 57 patients (10.3%); of these, 33.3% (19) had tuberculosis. Median hospital stay was 14 days (IQR 8–23) and 14.3% (79) of patients required re-admission to the hospital. Adverse events to medications received during the hospital stay were reported for 9.6% (n = 57) of the patients. Nosocomial infections were reported for 27 patients (4.9%).

Global mortality was 5.4% (30/551 patients). Of the patients who died, 70% (n = 21) had a previous diagnosis of HIV, 52.4% (n = 11) were receiving ART, and 38.1% (n = 8) were being followed-up in an HIV program. 96.6% (n = 28) of the patients that died had less than 50 CD4 cells/μL, 80% (n = 24) had been admitted for an AIDS-defining illness, and 36.6% (11 patients) had two or more OIs (Table 3).

**Table 3:** Main findings of HIV patients in Medellín, Colombia from August 2014 to July 2015

Variable	Patients who died (n = 30) n (%)	Patients who survived (n = 521) n (%)
Age	41.5	39.3
Male	25/30 (83.3)	393/521 (75.4)
Diagnosis during the index admission	9/30 (30.0)	112/521 (21.5)
Previous HIV diagnosis^a^	21/30 (70.0)	409/521 (78.5)
Diagnosis 1–12 months	6/30 (20.0)	117/521 (22.5)
Diagnosis > 12 months	15/30 (50.0)	292/521 (56.0)
On antiretroviral therapy at admission^b^	11/21 (52.4)	262/409 (64.2)
Adherence^c^	5/18 (27.7)	194/354 (54.8)
CD4 T-cells count < 200 cells/mL^d^	28/29 (96.6)	352/521 (67.4)
AIDS-defining illness on admission	24/30 (80.0)	277/521 (53.2)
Patients with newly diagnosed TB	7/30 (23.3)	121/521 (23.2)
AIDS defining illness no TB	17/30 (56.6)	156/521 (29.9)
Two or more OIs	11/30 (36.6)	96/521 (18.4)
Non-AIDS-defining illness but related to HIV	2/30 (6.6)	33/521 (6.3)

^a^ ≥ 1 month previous to hospitalization

^b^In patients diagnosed with HIV before admission

^c^In patients who had abandoned ART or were receiving ART prior to admission

^d^One patient had no CD4 T-cells count

## Discussion

This study describes the clinical characteristics, main causes of hospitalization and mortality rates in adult patients with HIV in a developing country. Contrary to what has been reported in high-income countries where cardiovascular, hepatic or neoplastic etiologies not associated with AIDS are the main causes of hospitalization [15–18], we found OIs to be the main reason for admission and mortality. Of the 30 patients who died, 80% (24 patients) died due to the opportunistic infection that caused the admission.

In low and middle-income countries the majority of admissions are still due to AIDS-defining events; a significant proportion of patients get diagnosed with HIV during this index admission or die with a recent diagnosis [6, 19]. Differences in opportunities to have access to programs, provision of medication and a timely diagnosis have been shown to influence the outcome of patients [20]. In our study 78% of hospitalized patients had a known diagnosis of HIV, 60% were getting follow-up in an HIV program and close to 60% were on ART. However, 57% (155) of patients on ART did not have adequate immunovirologic control. Our findings parallel national surveillance data in reference to HIV epidemiology [21]. In 2016, of 73,465 HIV-patients, 91.5% had access to ART, and, nonetheless, only 57.5% had accomplished virologic suppression [21]. The reasons for this gap between access to care and immunovirologic control were not directly assessed by our study. Most of our patients had socio-economic conditions that could favor non-adherence to ART [22, 23]. Our findings, in alignment with national data, suggest public health measures focusing on strategies to improve adherence and retention in HIV programs to maximize the benefit of antiretroviral treatment.

Studies from other developing countries report high mortality rates in patients hospitalized with HIV compared to those of industrialized countries (up to 38% vs. 2.6%). Mortality is associated to advanced disease stages, severity of immunologic compromise (low CD4 count), presence of opportunistic infections, and lack of resources for care [6, 15]. Although Colombia is considered a low-middle income country, we found comparable hospital mortality rates (5%) to those of industrialized countries [15, 16, 18] despite opportunistic infections as the primary reason for admission and advanced disease stage. This discrepancy between high frequency of opportunistic infections and low mortality is unexpected and could possibly reflect gaps in the overall quality of acute versus long-term, ambulatory care. Whether the low mortality rate observed is due to the quality of care provided in high-complexity hospitals with highly trained staff and medical resources to care for these patients, remains to be proved. However, previous studies suggest that increased physician experience in caring for patients with AIDS improves survival [24–26].

Strengths of the study include that it is a large, multicenter study with prospective design. Patients that were not included were analyzed retrospectively, finding similar causes of admission to those reported for the entire cohort (Fig. 1). Among the weaknesses, researchers did not have access to outpatient medical records, so information about HIV history was obtained from patient interviews, which could be associated with memory bias. Because of the prospective nature of the study and the requirement for informed consent, patients with CNS involvement who were unable to consent and who lacked a family member capable to do so were excluded. We recognize that this behavior might have generated some selection bias in the study. Additionally, patients were cared for in reference hospitals in Medellín, institutions with highly specialized staff and advanced technological resources. This is not the case for many health institutions in Colombia nor perhaps for health institutions in other developing countries. Our data may not be generalized to smaller cities in developing countries with differ to what happens in other Colombian cities lower complexity care hospitals.

## Conclusion

Opportunistic infections continue to be de the leading cause of hospitalization in adult HIV patients in Colombia. The gap between access to care and treatment and treatment goal achievement reflect possible programmatic limitations. Despite this, mortality rates observed were low, similar to those described for high-income countries. Our findings suggest focusing on measures to improve retention in care, ensure adherence and improve the opportunity for diagnosis, could have a long-term positive impact on the incidence of opportunistic infections and hospital admissions.

## Abbreviations

AIDS: acquired immunodeficiency syndrome
ART: antiretroviral therapy
HIV: human immunodeficiency virus
ICU: intensive care unit
MAC: Mycobacterium avium complex
OI: opportunistic infections
